# Health-related Behaviors in Pregnancy: A Key to Achieve Better Outcomes

**DOI:** 10.1055/s-0040-1708094

**Published:** 2020-03

**Authors:** Fernanda Garanhani Surita, Daiane Sofia Morais Paulino, Maira Pinho-Pompeu

**Affiliations:** 1 Department of Obstetrics and Gynecology, Universidade Estadual de Campinas, Campinas, SP, Brazil

In the last decades, changes in disease patterns from infectious to chronic diseases have made health-related behaviors become critical to the public's health and well-being.^1^ In pregnancy, similarly, the adoption of health-related behaviors has been recognized as a powerful element to minimize the occurrence of adverse maternal and perinatal outcomes and, consequently, improve woman and neonate's health. Moreover, it is well established that the intrauterine environment, which the fetus is exposed to, impacts not only the neonatal outcomes, but also program a susceptibility to long-term metabolic disease development.^2^

Gochman^3^ defined health behavior as “those personal attributes such as beliefs, expectations, motives, values, perceptions, and other cognitive elements; personality characteristics, including affective and emotional states and traits; and overt behavior patterns, actions, and habits that relate to health maintenance, to health restoration, and to health improvement.” Besides that, health-related behaviors are part of lifestyle and comprise modifiable health factors. Here, our aim was to discuss health-related behaviors involving diet, physical exercise (PE), healthy weight maintenance, stress management, sleep time, low alcohol intake, and non-smoking in the context of prenatal care.

Studies have shown that maternal dietary behavior modulates epigenetic alterations that impacts on offspring outcomes throughout childhood and can persist into adulthood. Thus, optimizing the nutrition in the first 1,000 days of life is opportune to prevent and reduce the risk of developing chronic diseases in the future.^4^ In this sense, a health pattern of consumption, such as in the Mediterranean diet (rich in fruits, vegetables, lean proteins, whole grains, omega 3 fatty acids and monounsaturated fats), is associated with lower risk of premature birth and newborn small for gestational age (SGA). In contrast, the western dietary pattern, characterized by high consumption of saturated fat, sugar, and salt is correlated with a significant increase in adverse neonatal outcomes.^5^ With regard to maternal health, a healthy dietary pattern was associated with lower risk of gestational diabetes mellitus (GDM)^6^ and hypertensive diseases^7^ and was protective against inadequate gestational weight gain (GWG).^8^ According to a dietary guide for the Brazilian population, a healthy eating pattern should be based on the predominant consumption of fresh and minimally processed foods, decreased consumption of processed foods, and avoiding the consumption of ultraprocessed foods.^9^ Thus, guiding an adequate dietary pattern in pregnancy according to the degree of food processing may be a new approach for nutrition care to prevent or minimize the occurrence of unfavorable pregnancy outcomes.

A growing body of evidence shows that the practice of PE during pregnancy reduces the risk of developing GDM, preeclampsia, and hypertensive disorders, in addition to the fact that PE prevents excessive GWG, cesarean section, and postpartum depression.^10^ ^11^ Despite the benefits of PE during pregnancy, it is described that the majority of pregnant women do not engage in any type of PE and tend to decrease the intensity or level of physical activity in the gestational period.^12^ The literature shows that, in Brazil, ∼ 20% of pregnant women^12^ follow the current recommendations for the practice of PE, which propose the weekly accumulation of at least 150 minutes of moderate intensity aerobic physical activity, at least 3 times per week.^13^

The PE and dietary patterns have been recognized as effective interventions for weight maintenance and obesity control.^14^ ^15^ In pregnancy, however, the efforts to maintain a healthy weight are not directed towards weight loss, but towards stimulating adequate GWG. To date, there is no guideline for GWG based on the Brazilian population, and, therefore, the current recommendation follows the guidelines proposed by the Institute of Medicine (IOM), which are formulated as a range for each category of pregestational nutritional status (according to body mass index [BMI]) and according to the gestational period.^16^ However, just monitoring the weight at prenatal care visits is not sufficient to meet the weight gain recommendations.^17^ The preventative measures to avoid inadequate GWG are to instruct pregnant women regarding nutrition, physical activity, and weight gain during prenatal care.^18^ Studies have shown that excessive GWG increases the risk of GDM, hypertensive diseases of pregnancy, cesarean section, postpartum weight retention, obesity, and future metabolic disorders.^19^ ^20^ Besides that, excessive GWG is correlated with macrosomia and childhood adiposity.^20^ ^21^

The maternal psychological state has also been linked with pregnancy outcomes. High maternal stress was associated with increased risk of preterm birth and low birth weight,^22^ chronic immune diseases, obesity, and metabolic disorders.^23^ Furthermore, prenatal maternal stress was associated with the adoption of unhealthy behaviors^24^ and adverse maternal outcomes, such as hypertensive disorders^25^ and postpartum depressive symptoms.^26^ Of note, health practitioners involved in perinatal care should be able to identify maternal distress, even in those women with a subclinical level, and provide appropriate support for women by developing positive coping strategies as well as ensuring that women receive a social support.^27^

In the last decade, the number of studies evaluating the relationship between quality and time of sleep with maternal and perinatal outcomes has grown. Thus, observational studies and systematic reviews have shown that poor sleep quality can predict the duration and type of delivery,^28^ reduce the quality of life and increase the risk of gestational hypertension, GDM, and postpartum depression symptom severity.^29^ ^30^ ^31^ In addition, sleep duration and sleep quality can affect neonatal outcomes such as birth weight and Apgar scores.^32^ These findings, however, lead us to the need to implement strategies for assessment and monitoring of sleep pattern during prenatal care. The most common methods for assessing sleep quality are actigraphy, polysomnography, and the Pittsburgh Sleep Quality Index (PSQI). Although the latter is a subjective method, it proved to be a good predictor of pregnancy complications.^33^ One of the challenges in improving sleep quality is that sleep quality improvement drugs are often unsafe during pregnancy.^34^ In this sense, effective and safe non-pharmacological strategies for improving sleep quality should be adopted during prenatal care.

The use of psychoactive substances, like alcohol, tobacco, and illicit drugs, in the gestational period is also commonly associated with worse maternal and perinatal outcomes and affect child development.^35^ Thus, aiming to avoid negative consequences, as fetal alcohol spectrum disorders, alcohol intake is discouraged, and, so far, no safe amount of alcohol has been established. Thereby, the current recommendations advocate abstinence among pregnant women and those trying to get pregnant.^36^

Smoking during pregnancy, as well as illicit drugs use, is associated with higher risk of preterm delivery, abortion, intrauterine growth restriction, and premature placental abruption.^36^ Thus, tobacco use is not safe during pregnancy. It is important to highlight that the adverse impacts of tobacco are not limited to active maternal use but also to passive exposure to tobacco.^37^ As part of a positive pregnancy experience, the World Health Organization (WHO) recommends health-care providers explain the dangers of tobacco use (past and present) for the pregnant women and about their exposure to second-hand smoke during every antenatal care visit. Besides that, whenever necessary, pregnant women should be advised and receive psychosocial interventions for tobacco cessation.^37^

Given the above, it is evident that the adoption of health-related behaviors during pregnancy is critical for promoting public health. In addition, a multiprofessional team is fundamental for improving the care of pregnant women. Antenatal care should also address the assessment, encouragement, and monitoring of the adoption of health-related behavior to optimize maternal and neonatal outcomes in both short and long term.
